# Occurrence and Distribution of *Fusarium* Communities in the Root Zone in a Post-Bog Permanent Meadow in Relation to Mineral Fertilization and Growing Seasons

**DOI:** 10.3390/pathogens11030341

**Published:** 2022-03-11

**Authors:** Teresa Korniłłowicz-Kowalska, Bernadeta Wojdyło-Kotwica, Justyna Bohacz, Michał Możejko

**Affiliations:** 1 Department of Environmental Microbiology, Faculty of Agrobioengineering, University of Life Sciences in Lublin, 20-069 Lublin, Poland; teresa.kornilowicz@up.lublin.pl (T.K.-K.); michal.mozejko@up.lublin.pl (M.M.); 2 Alab Plus Research Laboratory, 05-220 Zielonka, Poland; bernadeta.wojdylo.kotwica@gmail.com

**Keywords:** *Fusarium*, root zone, clovers, grasses, permanent meadow, peat-muck soil

## Abstract

The present study is the first report of a detailed analysis of the frequency of *Fusarium* and genera related to *Fusarium* colonizing the root zone of clovers and grasses growing in a permanent meadow established on peat-muck soil in a post-bog habitat. The isolation of fungi was carried out on the Nash and Snyder medium with the plate dilution method. The taxonomic identification of the collection of pure fungal cultures was based on morphological features revealed by macroscopic and microscopic observations. The species dominance coefficients, Marczewski–Steinhaus and Simpson species diversity index were calculated. Eight *Fusarium* complexes were distinguished. The distribution of the *Fusarium* population was uneven, which was generally reflected in a higher frequency of the *F. oxysporum* species complex in the clover root zone and *M. nivale*, *F. avenaceum* from the *Fusarium tricinctum* species complex, and *F. culmorum* from the *F*. *sambucinum* species complex in the grass root zone. The highest similarity of fungi was determined in the rhizoplane and the endorhizosphere. The highest species diversity and the highest population size were determined in the rhizosphere soil. The fertilization treatment reduced the growth rates in the *Fusarium* sensu lato and in genera related to *Fusarium*, as evidenced by the decrease in the total abundance and species richness. The root colonization by the *Fusarium*, especially the *F. oxysporum* species complex, was not accompanied by plant pathologies, which suggests a saprotrophic and endophytic rather than parasitic character of the relationships with the plant host.

## 1. Introduction

Drainage treatments carried out in the 1950s and 1960s in Poland resulted in the transformation of over 800 thousand ha of peat bogs into grasslands [1]. The reduction or disturbance in the natural moisture of peat soils resulted in the termination of the peat-forming process and, thus, the induction of a number of transformations generally referred to as muck-forming processes. Consequently, this process led to the formation of peat-muck soils. Permanent sodding, i.e., covering such soils with grass-dominated vegetation, and the use of such areas as meadows or pastures, is regarded as the most rational land-use practice, also in terms of the protection of these soils. Moreover, compact vegetation cover composed of permanent herbaceous plants such as grasses and legumes determines the specificity of meadows as a biotic formation. Little is known about the life and activity of microorganisms in soils and the root zone of the vegetation of permanent grasslands in post-bog habitats [2,3,4]. Mycological analyses of peat-muck soils were carried out by Tyszkiewicz [5,6,7,8]. However, the studies were conducted by the author in natural habitats in protected areas. The first more detailed data on the mycobiotic status of peat-muck soils used agriculturally as meadows were presented in our earlier studies [9,10,11]. The investigations were focused on the occurrence of spores of endomycorrhizal fungi and VA mycorrhiza. They also involved the characterization of saprotrophic fungal communities in the root zone of grasses and clovers of a permanent meadow established in a post-bog habitat. The present study shows the results of research on the presence of fungi from the *Fusarium* species complex and the sister group of *Fusarium* in this habitat. These fungi are associated primarily with agroecosystems and their specific microorganisms [12]. Previous investigations of the ecology of *Fusarium* in arable mineral soils [13,14,15,16,17,18] demonstrated the “affinity” of the genus to the roots of crop plants and its preference for acidic soils with a low level of clay minerals. These fungi are regarded as playing an important role in the functioning of plant communities and the maintenance of biological activity in cultivated soils. This is associated with three factors: (1) phytopathogenic *Fusarium* strains, often specific to certain plant hosts in multi-species herbaceous communities, can reduce their diversity [19]; (2) non-pathogenic strains in *Fusarium* populations can reduce the activity of pathogenic strains, thus contributing positively to plant health [20]; and (3) *Fusarium* fungi are ubiquitous and capable of saprotrophic competition for various C and energy sources, e.g., the degradation of the lignocellulosic complex, thus participating in nutrient cycling in soil [21]. Organic soils, e.g., peat-muck soils, exhibit a high abundance of lignocellulose-rich plant residues (dying sward composed of the roots, runners, and rhizomes of herbaceous plants).

Currently, there are scarce data on the presence of the *Fusarium* and genera related to *Fusarium* in the peat-muck soils of meadows [10]. There is no sufficient information on the distribution and activity of individual species of these fungi in the root system of meadow sward. To supplement the knowledge in this field, the present study provides a comprehensive characterization of *Fusarium* sensu lato and genera related to *Fusarium* in the root zone of clover–grass sward from a permanent meadow in a post-bog habitat. The investigations consisted of the determination of the total abundance, the richness and frequency of individual species, and the similarity and diversity of *Fusarium* and genera related to *Fusarium* colonizing the rhizosphere soil and the surface and cortex layer of clover and grass roots, taking into account the effect of NPK (nitrogen, phosphorus, potassium) fertilization and the vegetation season.

## 2. Results

### 2.1. Numbers of Fungi Isolated on the Nash and Snyder Medium

The *Fusarium* sensu lato and other genera of fungi were identified on the Nash and Snyder medium [22]. The identification of all pure cultures growing on this medium indicated the presence of approx. 80% of the *Fusarium* sensu lato (i.e., *Fusarium*; *Fusicolla aquaeductuum* and *Fusicolla merismoides*—formerly *Fusarium aquaeductuum* and *Fusarium merismoides*; and *Microdochium nivale*—formerly *Fusarium nivale*), with a small number of genera related to *Fusarium*, i.e., *Cylindrocarpon*/*Ilyonectria*. The other fungal genera were not investigated in this study.

**Figure 1:**
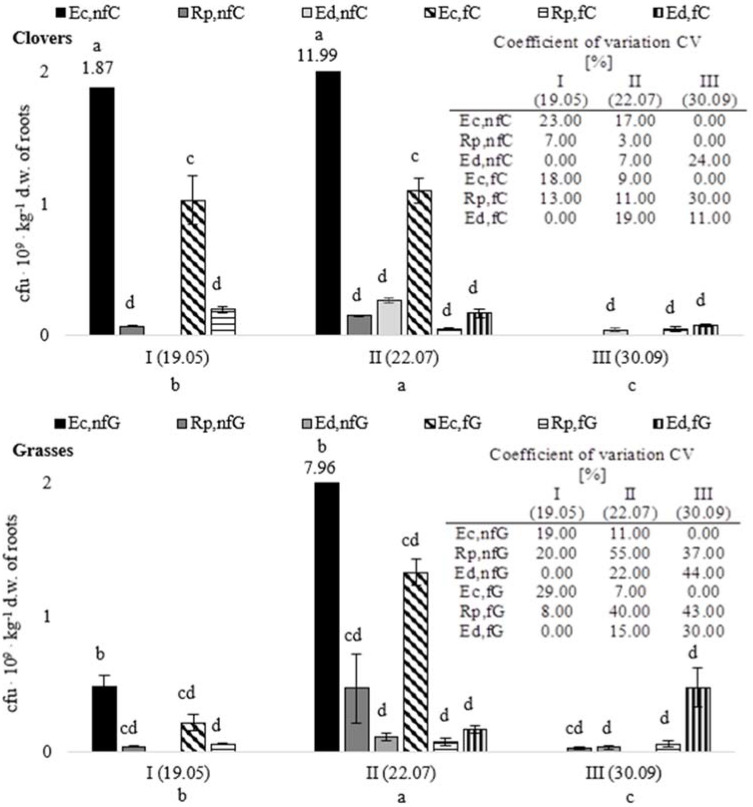
Numbers of fungi from the clover and grass root zone growing on the Nash and Snyder medium (cfu 10^9^ kg^−1^ d.w. of roots). Ec,nfC—ectorhizosphere of non-fertilized clovers; Rp,nfC—rhizoplane of non-fertilized clovers (wash 3); Ed,nfC—endorhizosphere of non-fertilized clovers; Ec,fC—ectorhizosphere of fertilized clovers; Rp,fC—rhizoplane of fertilized clovers (wash 3); Ed,fC—endorhizosphere of fertilized clovers; Ec,nfG—ectorhizosphere of non-fertilized grasses; Rp,nfG—rhizoplane of non-fertilized grasses (wash 3); Ed,nfG—endorhizosphere of non-fertilized grasses; Ec,fG—ectorhizosphere of fertilized grasses; Rp,fG—rhizoplane of fertilized grasses (wash 3); Ed,fG—endorhizosphere of fertilized grasses; AI, AII, AIII—analyses I (19.05), II (22.07), III (30.09); the same letters (a, b, c, d) indicate means that do not differ significantly from each other (at the significance level α = 0.05). However, the means of the compared pairs with different letters (e.g., a and b) differ significantly (at the same significance level α = 0.05).

The mean numbers of root zone-colonizing fungi isolated on the Nash and Snyder medium were 7.4 × 10^7^–4.6 × 10^9^ cfu × kg^−1^ d.w. of roots. The numbers of the *Fusarium* sensu lato and *Cylindrocarpon*/*Ilyonectria* were estimated at 5.92 × 10^7^–3.68 × 10^9^ cfu × kg^−1^ d.w. of roots. In the case of the rhizosphere soil (ectorhizosphere), the numbers were in the range of 2.2 × 10^8^–12.0 × 10^9^ cfu × kg^−1^ d.w. of roots. The surface of the roots (rhizoplane) and the root interior (endorhizosphere) were colonized by 9- to 25-fold smaller fungal communities, i.e., 2.5 × 10^7^–4.7 × 10^8^ cfu × kg^−1^ d.w. of roots and 3.2 × 10^7^–4.7 × 10^8^ cfu × kg^−1^ d.w. of roots (Figure 1). The numbers of fungi from the grass root zone growing on the Nash and Snyder medium were characterized by higher variability than the numbers of these fungi colonizing the clover root zone. This is evidenced by the higher values of the coefficients of variation CV, i.e., 0–55% and 0–30%, respectively. Higher CV values, indicating a large variability in the number of *Fusarium*, were recorded in July and September. Lower CV values, indicating lower variability, were noted in the studied group of fungi in spring (May) (Figure 1).

Significant differences in the level of numbers of ectorhizosphere fungi were noted in each of the four analyzed plant combinations. The most intense growth of these fungi was noted in the ectorhizosphere of the non-fertilized clovers. A high abundance of the fungi was also found in the ectorhizosphere of the non-fertilized grasses, although it was significantly lower than in the former microenvironment. In turn, the lowest (but similar) values, in comparison with the non-fertilized clovers and grasses, were recorded for fungi from the ectorhizosphere of the fertilized clovers and grasses. In terms of the growing season, a significant increase in the number of *Fusarium* sensu lato and the genera related to *Fusarium* was determined in July in both grass fertilization variants. An opposite effect (reduced abundance) was noted in the non-fertilized clover group (Figure 1).

In contrast to the ectorhizosphere, the mean of the numbers of fungi collected from the surface of plant roots from the analyzed combinations and cultured on the Nash and Snyder medium did not differ significantly, except for the non-fertilized grasses, where higher abundance of these fungi was observed (Figure 1). In terms of the seasonal dynamics, a significant increase in the number of the fungal groups was observed in summer (analysis II). Additionally, there were no significant differences between the mean of the number of the *Fusarium* sensu lato + *Cylindrocarpon*/*Ilyonectria* in the experimental variants (non-fertilized and fertilized clovers and grasses) in the case of the endorhizosphere. With regard to the dynamics of the seasonal abundance, there was a significant increase in the population of these fungi in autumn, i.e., in September (analysis III), in the endorhizosphere of the fertilized grasses. A reverse trend was observed in the other combinations of plants (Figure 1).

### 2.2. Fusarium and Genera Related to Fusarium

In total, 2210 isolates growing on the Nash and Snyder [22] medium were obtained from the root zone of clovers and grasses in all four grassland plant combinations. The subsequent part of the study was only focused on the analysis of fungi of the *Fusarium* sensu lato and genera related to *Fusarium* (*Cylindrocarpon/Ilyonectria)* due to the relatedness and similarity of these fungi and the preferences for the same growth substrates [23].

The species identification in the pure culture collection (Table 1 and Table 2) demonstrated that the root zones of all four combinations of plants were colonized by eight *Fusarium* groups. The *Fusarium oxysporum* species complex (FOSC) was classified according to the adopted frequency scale as a numerous species, with an average overall frequency in the plant root zone of 44.1% of the total *Fusarium* (Table 2). Within the other seven *Fusarium* groups, very frequent (range 11–25%) or frequent (range 6–10%) groups were the *Fusarium tricinctum* species complex (FTSC) and the *Fusarium sambucinum* species complex (FSAMSC). *Microdochium nivale* was a very frequent species (14.7%). In turn, FSAMSC with *F. sambucinum* and *F. graminearum*, FLSC with *F. lateritium*, and FFSC with *F. sacchari* were classified as rare species (1–5%). The other species were classified as sporadic (<1%). In total, *Cylindrocarpon* species and the *Ilyonectria radicicola* complex with *Cylindrocarpon/Ilyonectria destructans* accounted for less than 2% of the entire number of the *Fusarium* sensu lato plus the genera related to *Fusarium*, i.e., *Cylindrocarpon/Ilyonectria* isolates (Table 1 and Table 2). It was observed that some species, e.g., *F. tricinctum* from FTSC, were only present in the root zone of the non-fertilized clovers, while *F. merismoides* and *C. magnusianum* colonized the fertilized clovers (Table 2). The fertilization had no effect on the frequency of the analyzed *Fusarium* and genera related to *Fusarium*, as evidenced by the absence of significant differences between the fertilized and non-fertilized clovers and grasses (Table 2).

### 2.3. Distribution of the Fusarium and Genera Related to Fusarium in the Root Zone

#### 2.3.1. Ectorhizosphere and Endorhizosphere of Clovers

In total, 145 *Fusarium* strains were isolated from the ectorhizosphere of both the clover fertilization variants, representing seven species in the non-fertilized combination and six species in the fertilized plants (Supplementary Table S1). As indicated by the calculated species dominance coefficients (Table 3 and Table 4), *F. oxysporum* from FOSC was the most numerous species in both microenvironments. The species was more abundant in the rhizosphere soil of the fertilized (D = 68%) than non-fertilized (D = 41.4%) clovers. An especially high increase in the frequency of this species, i.e., 82.4% and 100%, respectively, was noted in July (analysis II). *F. avenaceum* from FTSC (non-fertilized plants) and *F. sporotrichioides* from FSAMSC (fertilized plants) were representatives of the *Fusarium* species complex reported in the clover ectorhizosphere most frequently (Supplementary Table S1, Table 3).

In both fertilization variants, the *F. oxysporum* species complex population substantially exceeded 50% of the total analyzed group of fungi in the endorhizosphere of the clover plants (Table 3). The population of this species accounted for 56.2% (non-fertilized plant) and 63.6% (fertilized plant) (Supplementary Table S1, Table 3). A lower but significant proportion was noted in the case of *Microdochium nivale* (13.5%) in the non-fertilized variant, and *F. avenaceum* from FTSC (16.9%) in the fertilized plants (Table 3). Additionally, it was found that the endorhizosphere of the non-fertilized clovers was colonized by a twofold higher number of species than the endorhizosphere of the fertilized clovers (10 and 5 species, respectively) (Supplementary Table S1).

#### 2.3.2. Rhizoplane of Clovers

In total, 796 fungal isolates were detected on the surface of clover roots, which were washed three times (wash 1, 2, and 3) to yield three fractions (II, III, IV) (Supplementary Table S2). Fraction IV from wash 3 was regarded to be the most representative fraction. In this material, within the 8 *Fusarium* species complexes, 14 species were identified, including 8 species detected after wash 3 with dominance of *F. oxysporum* from FOSC (D = 44.9%) (Table 3, Supplementary Table S2). *M. nivale* (D = 20.4%) was found to be the subdominant species. The mineral fertilization strongly stimulated the growth of the *F. oxysporum* species complex, as in the rhizosphere. The mean of the species dominance index was approximately 80%, or even 85.2% at term I (May). In turn, the fertilization reduced the *M. nivale* population size (D = 10.8%) (Table 3).

#### 2.3.3. Ectorhizosphere and Endorhizosphere of Grasses

The ectorhizosphere of the grasses differed in the structure of the *Fusarium* sensu lato and genera related to *Fusarium* from that of the clovers (Table 3 and Table 4). The non-fertilized combination was found to comprise nine species, whereas seven species were identified in the fertilized variant (Supplementary Table S3, Table 3).

*F. culmorum* (D = 38.5%) was the dominant species in the non-fertilized variant within FSAMSC, and *F. avenaceum* within FTSC (D = 27.8%) dominated in the fertilized group. The subdominant species were represented by *F. avenaceum*, *F. oxysporum*, and *F. sambucinum* within the FTSC, FOSC, and FSAMSC complexes, respectively, in the non-fertilized grasses, and *F. sporotrichioides*, *F. oxysporum*, and *F. lateritium* in the fertilized combination from FSAMSC, FOSC, and FLSC (Table 3).

Higher numbers of isolates were detected in the interior of the grass roots (endorhizosphere) in the non-fertilized and fertilized variants than in the rhizosphere soil (ectorhizosphere) (Supplementary Table S3). The quantitative relationships between the *Fusarium* differed as well. The *F. oxysporum* species complex accounted for the highest percentage in both combinations (non-fertilized and fertilized) (D = 61.4% and 54.0%, respectively). *Ilyonectria* *destructans* within the *Ilyonectria radicicola* complex was classified as a very frequent species (17.1%) in the endorhizosphere of the non-fertilized grasses. The endorhizosphere of the fertilized grasses was characterized by a high abundance of *F. culmorum* from FSAMSC (20.6%) and a lower proportion of *F. avenaceum* from FTSC (14.3%) (Table 3).

#### 2.3.4. Rhizoplane of Grasses

The total number of fungal strains isolated from the surface of the grass roots (rhizoplane) in both fertilization variants amounted to 445, which was represented by 17 species (Supplementary Table S4). In the studied *Fusarium* sensu lato and genera related to *Fusarium* in the non-fertilized grasses and fertilized grasses, *F. oxysporum* from FOSC (32.6% and 33.8%, respectively) and *M. nivale* (27.9% and 28.2%, respectively) were the dominant populations (Table 3 and Supplementary Table S4). In turn, *F. culmorum* from FSAMSC (11.6% and 11.3%) and *F. avenaceum* within FTSC (14.0% and 8.5%) were classified as very frequent species in accordance with the adopted frequency scale. The latter species was assigned as very frequent only in the non-fertilized variant (Table 3).

### 2.4. Similarity and Species Diversity of Fusarium and Genera Related to Fusarium in Root Zone of Clover–Grass Sward

The comparison of the number of *Fusarium* sensu lato and *Cylindrocarpon/Ilyonectria* colonizing the three microenvironments (ectorhizosphere, endorhizosphere, and rhizoplane) revealed a varied degree of species similarity, depending on the plant and fertilization combination as well as the term of the analyses. The mean values of the Marczewski–Steinhaus species similarity index (S) ranged from 17.8% to 65.7% (Table 5). The assessment of species diversity based on the Simpson index (taking into account the number and frequency of species) indicated significant differences in the quantitative relationships between the populations of the potentially phytopathogenic fungi: the total values of the Simpson index (D) ranged from 0.349 to 0.796 (Table 6). The comparison of the biota of these fungi in the root zone of all four plant combinations showed the lowest species diversity in the fertilized clovers.

#### 2.4.1. Clover Root Zone

In the group of clovers, the greatest similarity in the fungal species between the compared microenvironments was found in the non-fertilized variant. The rhizoplane and endorhizosphere were found to be most similar in terms of the composition of fungal species (value of coefficient S: 72.7%). In the case of analysis III, the similarity reached as much as 100%. In turn, the ectorhizosphere–rhizoplane and ectorhizosphere–endorhizosphere comparisons showed a decrease in the value of coefficient S, which indicates a decline in the number of common species in the subsequent periods of the growing season. Ultimately, coefficient S = 0% was obtained in analysis III, which reflected the absence of common species (Table 5).

In the fertilized variants, the degree of similarity of *Fusarium* and genera related to *Fusarium* between the compared microbiocenosis of the clover root zone was lower than in the non-fertilized variants and was at a similar level (S = ~30%). The highest value of coefficient S was obtained in the ectorhizosphere–endorhizosphere comparison (37.5% in total); however, it reached 50% in spring (analysis I) (Table 5).

The species diversity (Simpson species diversity indices: D) of the communities of the analyzed *Fusarium* and genera related to *Fusarium* colonizing the root zone of the fertilized clovers was found to decline. This effect was noted in all three microbiocenoses. This was particularly evident in the case of the rhizoplane (the lowest total coefficient D = 0.349). In turn, the Simpson species diversity indices in the *Fusarium* and genera related to *Fusarium* colonizing the non-fertilized clover root zone were generally high in each period of the vegetation season. At some analysis terms, very low or even no species diversity was noted, as in the case of the endorhizosphere (analysis II and III) (D = 0.112 and D = 0, respectively) (Table 6).

#### 2.4.2. Grass Root Zone

The comparison of both fertilization variants showed that the greatest species similarity (S) was between the rhizoplane and endorhizosphere in the grass root zone (44.4% and 55.6%, respectively). With regard to the individual terms of analysis, a gradual decline in species similarity was noted during the vegetation season in the root zone of the non-fertilized grasses (Table 5).

The total Simpson coefficients (D) for the individual microbiocenoses of the root zone of the grass combinations had similar values (Table 6). Slightly higher *Fusarium* sensu lato diversity was recorded in the ectorhizosphere and rhizoplane of the non-fertilized grasses than in the fertilized variant. In turn, an opposite phenomenon was evident in the populations of these fungi colonizing the endorhizosphere of these plants. Furthermore, it was noted that the diversity of the fungal populations in the endorhizosphere was lower than in the ectorhizosphere and on the root surface in this group of plants. In the case of the non-fertilized grasses, the Simpson index was only equal to zero in relation to the endorhizosphere (analysis II), which corresponded to the monoculture of the *F. oxysporum* species complex (FOSC) (Table 3).

### 2.5. Relationships between Frequency of Isolation of Fungi and Colonized Root Zone Microenvironment

The three χ^2^ tests (Table 7) indicated a significant relationship between the frequency of fungi and the colonized microenvironment. This is evidenced by the probability (*p* = 0.0001) calculated for the Pearson’s χ^2^ test and the χ^2^ maximum likelihood (ML) tests. The correlations are reflected by the values of the following indices: ϕ = 0.43; C = 0.40, and V = 0.22. The data from the contingency table demonstrated that the frequency of *F. oxysporum* from FOSC was correlated with each of the three analyzed root zone microenvironments (ectorhizosphere, rhizoplane, and endorhizosphere). However, this species was most strongly associated with the rhizoplane (22.5%) and the endorhizosphere (20.5%). A relatively high prevalence of *F. avenaceum* from FTSC and *Microdochium nivale* was noted as well. The largest population of the *Cylindrocarpon/Ilyonectria destructans* was detected in the rhizoplane (7.8%) (Table 7).

## 3. Discussion

### 3.1. Abundance of Fusarium in Plant Root Zone

Fungi of the genus *Fusarium* are often recovered in grasslands [12,24]. They occur in this environment as saprotrophs, endophytes, and pathogens of meadow vegetation, i.e., grasses [25] and clovers [26,27]. They colonize non-rhizosphere and rhizosphere soil as well as the surface and cortex layer of roots [12,27,28,29]. As reported by Wilberforce et al. [12] and Yli-Mattila et al. [30], the presence of *Fusarium* in meadow soils is highly supported by agricultural land use. As demonstrated in our previous study (Korniłłowicz-Kowalska et al. [10]) on communities of culturable saprotrophic fungi of the root zone of grasses and clovers in a permanent meadow established in a post-bog habitat, the genus *Fusarium* was the third largest population (on average 10.4%) after *Trichoderma* (on average 60.5%) and *Penicillium* (11.3%). The co-dominance of *Fusarium* with *Trichoderma* and *Penicillium* in the root zone of plants (cereals) was also reported by Kurek et al. [31]. It was found in the present study that the number of fungi in the root zone of the clovers and meadow grasses reflected in the colony forming units (cfu) cultured on the *Fusarium* selective medium ranged from 6 × 10^7^ to 4 × 10^9^ cfu × kg^−1^ d.w. of roots (this corresponds to 60 thousand to 4 million cfu × g^−1^ d.w. of roots). These values are higher than the *Fusarium* population density determined in mineral soils (sandy soil and loamy soil; 1.3 × 10^4^ and 8 × 10^3^ cfu × g^−1^ d.w. of soil, respectively) by Korniłłowicz [14]. The high abundance of the *Fusarium* and genera related to *Fusarium* in the root zone of the meadow phytocoenoses in the post-bog habitat was facilitated by the properties of this biotope—in particular, the high content of organic matter (over 50%), which is rich in easily available lignocellulose and non-cellulosic polysaccharides from grasses and other herbaceous plants. As reported by Wichern et al. [32], dead fragments of meadow sward, including roots, may account for up to 90% of rhizodeposits. Combined with the ability of *Fusarium* to degrade cellulose and other polysaccharides [15,21], this creates good nutritional conditions for the saprotrophic growth of these fungi. Abiotic factors supporting *Fusarium* growth in peat-muck soils include moisture (hydrogenic soils) and low pH. As reported by Strzelczyk [33], *Fusarium* and *Cylindrocarpon* colonize plant roots more effectively at 50% soil moisture and 70% water capacity than at 30% water capacity. The stimulation of *Fusarium* growth in strongly acidic meadow soils (mineral soils) was reported by Dorenda [34]. The preference of *Fusarium* fungi for environments with elevated moisture is associated with their high- water requirements (water activity coefficient a_w_ = 0.90–0.95), which classifies these fungi in the group of tertiary colonizers, i.e., those with the highest requirements for water conditions [35]. In turn, *Fusarium*’s tolerance of acidic environments is associated with the release of acids as products of carbohydrate metabolism by these fungi, which is responsible for their resistance to acidification [36].

The greater fluctuations in the number of the *Fusarium* and genera related to *Fusarium* observed in the root zone of the grasses than in the root zone of the clovers (higher values of coefficients of variation CV in the grasses) most probably resulted from the differences in the root secretions of these plants. This assumption is supported by findings reported by Broeckling et al. [37], which proved the impact of root secretions on the development and structure of “root” fungi. In the light of this information and the results of the present study, we believe that grass root exudates, which are rich in easily available carbon sources (including sugars C_6_ and C_3_) and have a periodically changing quantitative and qualitative composition [38], stimulated the growth of *Fusarium* more efficiently than clover root secretions, which are rich in organic and mineral nitrogen compounds (symbiosis with *Rhizobium*) [32,38]. The weaker impact of the latter is associated with a narrower C:N ratio, while it is known that fungi require large amounts of organic C for mycelial growth.

The dynamics of fungal growth in the root zone of both studied groups of plants, in accordance with the seasonal fluctuations in the number of soil fungi [39], generally exhibited lower abundance in spring (May) and higher abundance in summer (July) or autumn (September). Similar seasonal fluctuations of *Fusarium* in meadow soils were reported by McMullen and Stock [17]. This effect was undoubtedly mainly associated with the diversified supply of rhizodeposits in the different seasons related to plant growth stages and soil temperature and moisture. Seasonal changes in *Fusarium* productivity in grassland soil were shown to be induced by fluctuations in the organic matter supply determined by changes in plant physiology during the vegetation season [17]. However, given the specificity of the climatic conditions prevailing in the study area, we assume that temperature is an important factor as well. Undoubtedly, after the period of winter stagnation and recurrent spring frosts, the impact of plant root secretions must have been weaker than during the full vegetation season characterized by an increase in air and soil temperature and sufficient soil moisture. Moreover, with a few exceptions such as *Microdochium nivale*, the optimum temperature for *Fusarium* growth exceeds 20 °C [23]. The great importance of habitat conditions for the number of fungi in peat soils was emphasized by Tyszkiewicz [6]. As reported by Bissett and Parkinson [40], soil moisture and temperature are the most important abiotic factors in the seasonal fluctuations in the soil fungal composition (including *Fusarium*).

The distribution of the *Fusarium* and genera related to *Fusarium* in the root zone of both groups of plants was uneven, which was reflected in their higher frequency in the rhizosphere soil than in plant roots (rhizoplane and endorhizosphere). It corresponded to the distribution of the total saprotrophic fungi in the root zone of the analyzed plants [10]. This was caused by the greater pool, variety, and availability of rhizodeposits in the rhizosphere soil, which may even represent 40% of assimilates deposited in roots, as shown by Lynch and Whipps [41]. The lower abundance of the *Fusarium* and genera related to *Fusarium* on the root surface and in the cortex layer was also related to the greater difficulties in the saprotrophic colonization of these microenvironments, e.g., plant defense mechanisms and the inaccessibility of organic matter as a fungal food source. Yuan et al. [42] have reported that the colonization of internal root tissues by saprophytic fungi requires a specific ability to penetrate and grow in this environment. In turn, Hoyos-Carvajal et al. [43] have demonstrated that only some strains in populations of saprotrophic rhizosphere fungi are adapted to the colonization of surface structures, intercellular spaces, and root cortex cells. Given the similar frequency of these fungi in the rhizoplane and endorhizosphere of the analyzed clovers and grasses, it can also be concluded that the conditions for the growth of the fungi in these microenvironments are similar.

It was found that mineral fertilization (NPK) reduced the overall number of *Fusarium* and related fungi in the root zone of the clover–grass sward, with a substantial reduction in the ectorhizosphere and endorhizosphere. This effect should be attributed to the stimulation of the growth of antagonistic *Trichoderma* fungi in these environments, which was demonstrated in our previous study [10]. The stimulatory effect of mineral fertilization on *Trichoderma* growth in non-rhizosphere agricultural soil and the accompanying decline in *Fusarium* abundance, in contrast to variants with no mineral fertilization treatment, were reported in our other investigations [14]. The activation of *Trichoderma* growth induced by NPK fertilization of clover and grass sward was assigned by Korniłłowicz-Kowalska et al. [10] to an increase in the nitrogen-rich fraction in root exudates. As demonstrated by Wilberforce et al. [12], differences in soil nitrogen content are responsible for changes in the structure of root-colonizing fungal communities. The limitation of the intensity of *Fusarium* growth by antagonistic *Trichoderma* fungi may involve many mechanisms, e.g., competition for carbon and iron substrates, antibiosis, or mycoparasitism [44].

### 3.2. Species Richness, Similarity, and Diversity in Fusarium Communities Colonizing the Plant Root Zone

The root zone of the clovers and grasses in the permanent meadow established on organic peat-muck soil was colonized by the multi-species *Fusarium* and genera related to *Fusarium*. A majority (14) of the identified species exhibited low frequency, with the exception of a few species: the most abundant *F. oxysporum* from FOSC and three others, i.e., *Microdochium nivale*, *F. avenaceum* from FTSC, and *F. culmorum*, from FSAMSC. The similarity of the fungal species between the analyzed environments was not high, although it exceeded 50% in the case of some species.

A high *Fusarium* diversity in the soil and the root zone of clovers and grasses in meadow cultivation (loamy sand) was also reported by McMullen and Stock [17] and LeBlanc et al. [45]. Our observations are particularly consistent with the results of research conducted by McMullen and Stock [17], who found that communities of *Fusarium* fungi in multi-species meadow plant communities exhibit high richness (20 species) and varied species similarity and population size. In the present study, there were greater numbers of common species (higher species similarity coefficients) in the rhizoplane–endorhizosphere zone than in the rhizosphere soil–root group (surface and interior). This phenomenon was observed in both the grasses and the clovers, which indicates selection within the *Fusarium* communities. The selection takes place during the “re-settling” from the ectorhizosphere to the root surface and next to the root cortex. Greater similarity of the living conditions of the mycobiota was found between the rhizoplane and the endorhizosphere than between the ectorhizosphere (soil) and the root. This promotes greater species similarity in fungi colonizing the roots than the soil.

There was a decrease in the number of common species colonizing the fertilized plants, especially in the rhizosphere soil and the root (surface and cortex layer). A similar phenomenon was observed during the changes in the growing season, which was reflected in the highest species similarity coefficients in spring (May—analysis I) and their lower values in summer (July—analysis II) and sometimes at the end of the vegetation season (September—analysis III). It seems that the key role in the reduction of similarity between the *Fusarium* sensu lato and genera related to *Fusarium* in the middle of the growing season was played by changes in the quantitative and qualitative composition of rhizodeposits, i.e., an increase in the concentration of soluble fractions in relation to the insoluble lignocellulose. The greater abundance of the insoluble fraction in spring (accumulation of dead organic matter) enriched the population spectrum, with species exhibiting more diverse physiological abilities. In turn, the predominance of the simple soluble sources of C and energy in the full vegetation season contributed to the selection of a narrower spectrum of populations, which simultaneously exhibited stronger saprotrophic competition activity. This is related to the fact that, although soluble carbon and energy sources are available to all saprotrophs, fungi with more potent competition mechanisms, e.g., rapid growth or secretion of antimicrobial metabolites, assimilate the sources more readily. This phenomenon can be observed during the colonization of complex organic matter by fungal consortia [46]. It consists of the succession of physiologically diverse groups of fungi, among which communities with a broad substrate spectrum (ubiquitous fungi) exhibit higher species richness than the so-called sugar fungi utilizing simple soluble sources of C and energy [46]. This explanation may also be confirmed by the dynamics of the Simpson species diversity coefficients in terms of the number of the fungi in the root zone of the analyzed plants. In both groups of plants, the Simpson coefficient in the *Fusarium* and genera related to *Fusarium* was higher in spring and lower in summer. This effect was caused by the reduction of the number of species and the selection of a few populations in the middle of the vegetation season, including the monoculture form (endorhizosphere). The mineral fertilization contributed to changes in rhizodeposits, i.e., an increase in the concentration of nitrogen-containing compounds [38], which may have resulted in the selection of only some *Fusarium* populations preferring a narrower C:N ratio. One of such species is *F. oxysporum*, which can grow abundantly on animal-origin substrates [47]. Similar trends in the species similarity and diversity of saprotrophic fungi colonizing the rhizosphere soil and roots of meadow plants were reported in our earlier study, where mineral fertilization and the influence of the growing season were considered [10].

The present study showed four dominant populations in the multi-species *Fusarium* communities of the root zone of the clover–grass sward, i.e., the dominant *F. oxysporum* from FOSC (on average 44.2% of the total number of the *Fusarium* sensu lato and three co-dominant species: *M. nivale* (15%), *F. avenaceum* from FTSC (11.6%), and *F. culmorum* from FSAMSC (~9.15%)). In general, the *F. oxysporum* population colonized the clover root zone more potently, whereas *M. nivale*, *F. avenaceum* from FTSC, and *F. culmorum* from FSAMSC were more frequently identified in the grass root zone. Since the population size of fungi is an indicator of their activity [8], these four species can be regarded to have the greatest importance in the relationship between *Fusarium* and the root zone of the clover–grass sward in the post-bog habitat.

The high frequency and even dominance of *F. oxysporum* populations in soils and roots of meadow plants growing on mineral soils have been reported by many authors [12,17,45,48,49]. As indicated by the present results (χ^2^ tests), this species colonized the rhizoplane and endorhizosphere of both groups of plants most potently, with particular preference for the fertilized plants. The greater “affinity” of *F. oxysporum* to roots, especially the cortex, than to the ectorhizosphere of clover and meadow grasses in mixed cultivation on mineral soil was reported by Dorenda [13,34,50].

We believe that, in the absence of disease symptoms in the analyzed clover and grass plants, the present data indicate the endophytic character of the colonization of the roots of these plants by *F. oxysporum* from FOSC in peat-muck soils. This also suggests a biocontrol function of saprotrophic *F. oxysporum* strains from FOSC in meadow phytocoenoses in the post-bog habitat. It is known that pathogenic strains of *F. oxysporum*, i.e., *F. oxysporum* sp. *trifolii*, are one of the most dangerous fungal pathogens of clover [34], whereas non-pathogenic *F. oxysporum* strains are classified as antagonists of the pathogenic strains of this species [20,51]. The intense colonization of the endorhizosphere of the analyzed meadow plants by the saprotrophic *F. oxysporum* strains from FOSC can therefore be regarded as a protective factor against the fusariosis caused by pathogenic *F. oxysporum* strains. The mechanism of the antagonistic interaction between saprotrophic *F. oxysporum* strains and their pathogenic counterparts is based on, e.g., the competition for C and Fe sources, faster colonization of the root surface, and induction of plant resistance by endophytic strains [20,52]. As reported by Alabouvette [20], the intensity of the intraspecific competition in the *F. oxysporum* population (pathogenic and non-pathogenic strains) for carbon sources (glucose) depends on the availability of Fe and decreases when the concentration of the element decreases.

The present study shows that, among the three co-dominant species, *F. culmorum* from FSAMSC and *F. avenaceum* from FTSC mainly colonized the grass root zone, which was evident in all three microenvironments: the rhizosphere soil, the root surface, and the cortex layer. In the temperate climate zone, *F. culmorum* and *F. avenaceum* are pathogens causing root rot and stem rot in many crops, especially in cereals, but also in legumes and grasses. They can also be saprotrophs and endophytes [53,54,55,56]. *F. avenaceum* from FTSC is considered to be less pathogenic than other species in the *Fusarium* complex that cause cereal and grass diseases [26,30,48,57]. Non-pathogenic *F. culmorum* strains, likewise non-pathogenic *F. oxysporum* strains, may exhibit antagonistic activity against pathogenic strains causing head blight and root rot [53,55]. Our results are consistent with the findings of the limitation of the growth of *Fusarium* species in wetland soils subjected to mineral fertilization and located along tropical lakes [58,59]. In turn, our results differ from those reported for lacustrine soils by [60,61,62], who found that root colonization by *Fusarium* complex species, especially *F. oxysporum*, was accompanied by pathologies in plants. These authors showed that the cause of these changes was a fungus–bacterium complex comprising bacteria of the genus *Pectobacterium* and *Erwinia* in addition to *Fusarium* (*F. moniliformae*, *F. oxysporum*, and *F. solani*). As shown by Domsch et al. [23], *F. culmorum* and *F. avenaceum* are very often isolated from healthy plants. The absence of pathological symptoms in the grass roots analyzed in the present study, accompanied by the high proportion of *F. culmorum* and *F. avenaceum* in the *Fusarium* communities in the ectorhizosphere and their substantial abundance in the rhizoplane, prove the saprotrophic growth of these species in the rhizosphere and on the root surface. In their investigations of the antagonism between non-pathogenic *F. culmorum* strains and pathogenic *F. culmorum* strains, i.e., the causative agents of rye fusariosis, Kurek and Jaroszuk [55] showed that the antagonism was based on different capabilities of iron complexation. The non-pathogenic strains, in contrast to the pathogenic ones, produced Fe-complexing compounds, which is an indispensable factor for spore germination and root infection by pathogenic *F. culmorum* strains [55]. The lower abundance of *F. culmorum* and *F. avenaceum* in the endorhizosphere of the meadow grasses evidences the lower activity of the saprotrophic strains of these species inside the roots than on the outer surface and in the soil adjacent to the roots. The highest productivity of *F. culmorum* in the rhizosphere soil of the grasses was detected in the non-fertilized sward and in the root interior in the fertilized variant. The differences in the dynamics of the *F. culmorum* population growth in the root zone of the fertilized and non-fertilized grasses can be explained by the dynamics of the growth of the antagonist of these fungi, i.e., *Trichoderma* spp. This genus colonized the ectorhizosphere of the non-fertilized grasses and the endorhizosphere of the fertilized grasses less efficiently [10].

The third co-dominant species with *F. oxysporum* from FOSC, i.e., *M. nivale*, is commonly found in meadows and various types of grasses, especially in the temperate and cool climate zones, where it can cause root and seedling rot (so-called snow mold) in early spring. A high abundance of *M. nivale* was detected primarily on the root surface, with a greater proportion in the grasses (28%) than in the clovers (20%). This fungus was also found to colonize the root cortex in the analyzed plants, although less efficiently. In field experiments on spring barley, Perry [63] showed that *M. nivale* caused latent infections with no damage to plants. As suggested by the author, *M. nivale* may behave like an endophyte, and thus serve a protective function in the host plant. Therefore, the absence of visible infection symptoms in the analyzed clover and grass plants throughout the observation period may indicate the non-pathogenic nature of the *M. nivale* growth (as well as the other *Fusarium*) in the root zone of the meadow sward in the post-bog habitat.

## 4. Materials and Methods

**Figure 2:**
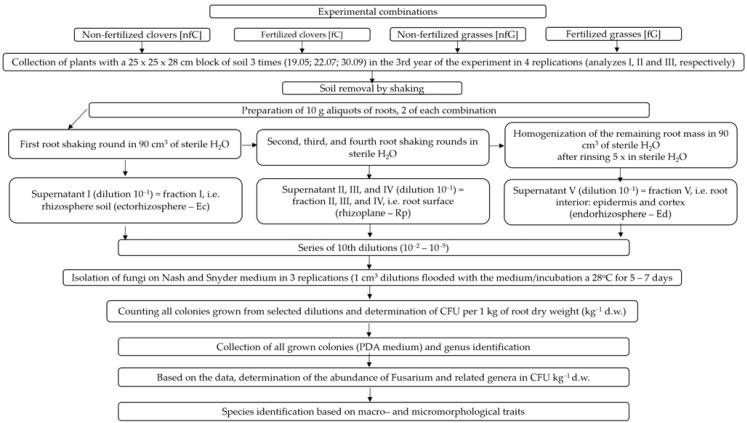
Scheme of the isolation, quantification, and identification of the *Fusarium* genus and related genera.

The scheme of the isolation, quantification, and identification of the *Fusarium* genus and related genera is presented in Figure 2.

### 4.1. Study Area

**Figure 3:**
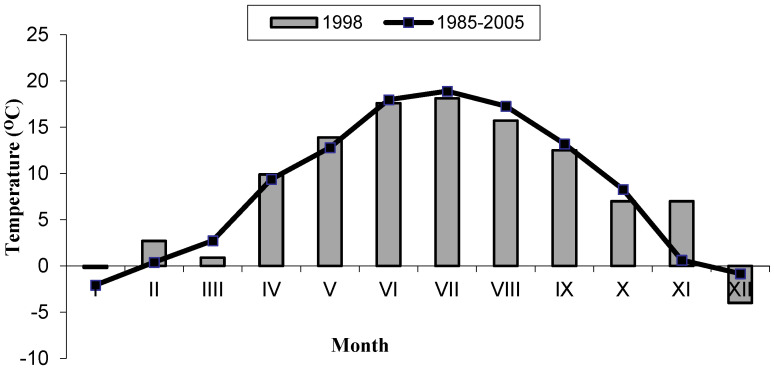
Average monthly air temperature in 1998 (data from the Uhnin Meteorological Station) vs. the historical period 1985—2005.

**Figure 4:**
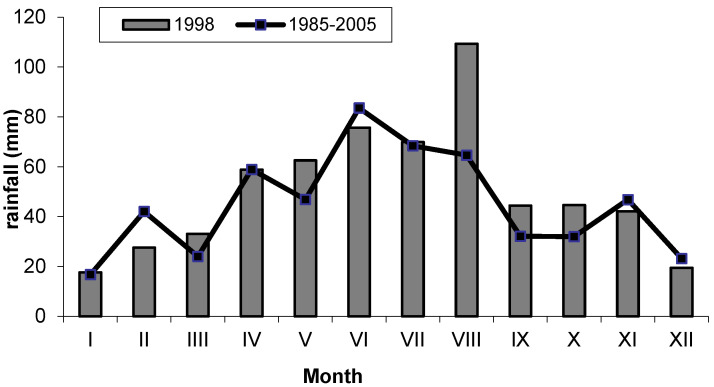
Rainfall sums in 1998 (data from the Uhnin Meteorological Station) vs. the historical period 1985—2005.

The study was carried out in an experiment established in June 1996 by the Department of Grassland and Landscape Management, University of Life Sciences, Lublin, at the Didactic and Research Station in Sosnowica (south-eastern Poland, Lublin Province), which is part of the Department of Grassland and Landscape Management, University of Life Sciences, Lublin. The meadows where the experiment was established are located between the Piwonia River (a tributary of the Tyśmienica River) and the Wieprz-Krzna Channel (51°31′ north latitude and 23°04′ east longitude). In 1964–1965, the meadows were drained and managed, which led to the transformation of the marsh habitat with peat-bog soils into a post-bog habitat with peat-muck soils [64,65]. The investigations were carried out in the third year of the experiment (1998). Some physical and chemical properties of the analyzed soil are presented in Table 8, and the weather conditions are shown in Figure 3 and Figure 4.

The experiment was conducted in a mixed block design in four replicates of 24 m^2^ plots. White clover (*Trifolium repens* L.), red clover (*Trifolium pratense* L.), blue grass (*Poa pratensis* L.), timothy (*Phleum pratense* L.), and cat grass (*Dactylis glomerata* L.) were sown on the plots. The experimental combinations with clover were denoted as C and those with grasses were marked with the letter G. In the mixture, they accounted for 17.5, 17.5, 35, 20, and 10%, respectively, at the sowing rates of 15, 21, 24, 18, and 21 kg × ha^−1^, respectively. The clover–grass sward was mown three times during the growing season, and the following NPK (N: nitrogen, P: phosphorus, K: potassium) fertilization rates (kg × ha^−1^) were applied: N: 40, P_2_O_5_: 80, and K_2_O: 120. The fertilizers were applied by hand evenly over the entire plot area. The nitrogen fertilization was applied in three doses (1/3 each) before the beginning of the vegetation season and after the first and second mowing event. The phosphorus fertilization was applied once in spring before plant vegetation, and the potassium fertilization was provided in two doses (1/2 each) in spring and after the second mowing event. Clover–grass sward without NPK fertilization served as a control. The fertilized (f) and non-fertilized (nf) variants with clover (C) and grasses (G) were denoted with fC, fG and nfC, nfG, respectively. In both plant combinations, the clovers and grasses exhibited no symptoms of disease in their aboveground parts and roots.

### 4.2. Isolation and Identification of Fungi

The study material consisted of plant roots collected three times: before the first (19 May), second (22 July), and third (30 September) mowing events in the phase of clover buds and grass flowering in the third year of the experiment. The analyses were denoted as follows: first term—AI, second term—AII, and third term—AIII. The plant material was collected and prepared as in Król and Kobus [66]. The plants were sampled together with a 25 × 25 × 28 cm block of soil adjacent to the root in four replications. The soil was shaken off the roots and, on average, 10 g samples were prepared (two from each combination). The root aliquots were shaken in 90 cm^3^ of sterile distilled water for 20 min. The supernatant constituted fraction I corresponding to the rhizosphere soil (ectorhizosphere—Ec), as specified in the methodology proposed by Król and Kobus [66]. The plant roots were flooded with distilled water and shaken again to obtain fractions II, III, and IV (wash 1, 2, and 3), which represented the root area (rhizoplane—Rp). The remaining root mass was washed and shaken five times, and then homogenized (10 min, 3000 rpm) on ice to obtain fraction V, representing the interior of the root (endorhizosphere—Ed) with microorganisms colonizing the epidermis and primary cortex [66]. All of the supernatants (fractions I–V) were the initial (stock) dilutions for the isolation of fungi of the *Fusarium* sensu lato and genera related to *Fusarium* (*Cylindrocarpon/Ilyonectria*).

The fungi were isolated on the Nash and Snyder [22] medium for the isolation of *Fusarium* species composed of (g × dm^−3^) peptone -15, KH_2_PO_4_ -1, MgSO_4_ × 7H_2_O-0.5, agar-20, PCNB (pentachloronitrobenzene)-1, streptomycin-300 mg × dm^−3^, and distilled H_2_O-1 dm^3^. The isolation was carried out using the plate dilution method. The cultures were incubated at 26 °C for 5–7 days in three plate replicates for each dilution. The number of fungi growing on the Nash and Snyder [22] medium was expressed in colony forming units (cfu) per g^−1^ of dry root weight, determined after drying at 105 °C. The number of *Fusarium* fungi was determined after the identification of the fungal genus and expressed as above. The genus and species composition of the fungi was determined in one randomly selected plate with at least 30 colonies (from three replicates for each series). When the number of colonies was smaller, two or three plates were analyzed to achieve a total of ≥30. All colonies were inoculated on glucose–potato broth slants. The taxonomic identification of the collection of pure fungal cultures was based on the morphological features revealed by macroscopic observations of the plates and slants and microscopic observations of the microcultures. The analyzed traits included colony morphology, mycelium color (obverse, reverse), substrate pigmentation, and the production and morphology of spores (macro- and microconidia) and chlamydospores. Biometric measurements were performed when necessary. *Fusarium* species were identified on the glucose–potato substrate (PDA) and Nirenberg agar (SNA) [56] (g × dm^−3^): glucose—0.2, sucrose—0.2, KH_2_PO_4_-1, KNO_3_-1, MgSO_4_ × 7H_2_O-0.5, KCl—0.5, agar-15, distilled H_2_O-1 dm^3^. Micromorphological observations were carried out with the use of a research microscope (Olympus BX11) equipped with a digital/CVIII4 camera coupled with a computer with an installed CellA program.

The systematic studies conducted by Domsch et al. [23], Kwaśna et al. [56], and Nelson et al. [62] were used to identify the genus and species of the isolated fungi.

The identified species of fungi were described as the *Fusarium* complexes based on their affiliation presented in the literature [65,66,67,68,69,70,71,72]. Eight *Fusarium* complexes with their assigned species were distinguished, i.e., the *Fusarium sambucinum* species complex ((FSAMSC) with *F. culmorum, F. graminearum, F. poae, F. sambucinum, F. sporotrichioides*), the *Fusarium tricinctum* species complex ((FTSC) with *F. avenaceum* and *F. tricinctum*), the *Fusarium incarnatum-equiseti* species complex ((FIESC) with *F. equiseti* and *F. incarnatum*), the *Fusarium oxysporum* species complex ((FOSC) with *F. oxysporum*), the *Fusarium fujikuroi* species complex ((FFSC) with *F. sacchari*), the *Fusarium solani* species complex ((FSSC) with *F. solani*), the *Fusarium lateritium* species complex ((FLSC) with *F. lateritium*), and the *Fusarium camptoceras* species complex ((FCAMSC) with *F. camptoceras*). Given the finding reported by Geiser et al. [73], i.e., “We see no benefit in splitting *Fusarium* in favor of competing names that are largely tied to rarely observed sexual” and the equivalence of the *Fusarium* and *Gibberella* names indicated by Lombard et al. [74] and Crous et al. [75], the identified species *Gibberella avenacea* and *Gibberella pulicaris* were assigned to FTSC and FSAMSC, respectively. The other strains were classified into genera related to *Fusarium*, i.e., the *Ilyonectria radicicola* complex according to Guan et al. [76] and the *Cylindrocarpon* and *Fusicolla* groups according to O’Donnell et al. [68].

### 4.3. Analysis of Results

A multivariate analysis of variance (ANOVA) was used to show significant differences in the number of fungi between the analyzed microenvironments (the ectorhizosphere, rhizoplane, and endorhizosphere) in the experimental plant combinations (non-fertilized and fertilized clovers and non-fertilized and fertilized grasses). A one-way ANOVA, followed by Tukey’s post hoc test, with a significance level of α = 0.05 was carried out to demonstrate the differences between the frequency of occurrence of *Fusarium* complexes, fungi related to *Fusarium*, and the non-fertilized and fertilized clovers and grasses using STATISTICA software ver.13.3 (StatSoft, Kraków, Poland). The significance of the differences between the means was assessed with a Tukey’s test at the significance level α = 0.05. This allowed the assignment of the experimental variants into statistically homogeneous groups and the determination of the smallest significant difference of means with Tukey’s honestly significant difference (HSD) tests [77]. The descriptive statistics involved the calculation of the means, standard deviations, and CV coefficients of variation as a measure of the random variability of the analyzed variables.

The analysis of multi-way contingency tables was performed to identify correlations between the frequency of the *Fusarium* and genera related to *Fusarium* occurrence and the colonized microenvironment (the ectorhizosphere, endorhizosphere, and rhizoplane) [77,78,79,80]. Due to the large variability of the experimental data, which did not allow the selection of one optimal statistical method, several calculations methods, i.e., three chi-square (χ^2^) tests for independence, were employed, including Pearson’s chi-square test, the chi-square maximum likelihood (ML) test, and the Mantel–Haenszel chi-square test. To interpret the results, it was assumed that when the calculated p (probability) is < α (at the significance level α = 0.05), there is a significant relationship between the analyzed variables. Moreover, to determine the strength of these relationships (the χ^2^ test only shows whether two variables are related to each other), c-Pearson’s contingency coefficients, Φ-Yule’s dependence coefficients (phi), and γ-Cramer coefficients were calculated. They have values from 0 (independence of variables) to 1 (close relationship of variables). The calculations and comparisons of the data were carried out with the use of the STATISTICA statistical package (Statsoft, Poland).

With regard to the analysis of the species composition in the fungal communities, the species dominance coefficients (D) were calculated using the following formula [81]:

D = 100 (S_a_:S)

where: Sa—sum of isolates of species a; S—sum of isolates of the studied group (ectorhizosphere, rhizoplane, endorhizosphere).

The species frequency was assessed using the scale proposed by Korniłłowicz-Kowalska et al. [10] for root zone fungi: <1% = sporadic, 1–5% = rare; 6–10% = frequent, 11–25 = very frequent, 26–50% = numerous, and >50% = very numerous.

The similarity between the analyzed fungal communities was determined with the use of the Marczewski–Steinhaus formula [82]:

S (1,2) = w/(a + b − w)

where: S—species similarity between two communities; a—number of species in community 1; b—number of species in community 2; w—number of species that are common to both communities. When both sets have all elements in common, the similarity S = 1, i.e., 100%. When both sets do not have common elements, S = 0.

The analysis of species diversity of fungal communities, taking into account the number of isolates (=number of records) of individual species in the analyzed microenvironments (ectorhizosphere, rhizoplane, endorhizosphere), was performed using the Simpson species diversity index (D) developed on the basis of the probability theory [83]:

$$D=1\;-\sum_{i=1}^{S}\left({\mathrm{pi}}^{2}\right)\;$$

where: pi—proportion of isolates (strains) of species “i” in a given fungal community, with pi = mi/N (mi—number of strains of species “i”); N—total number of strains. The values of the Simpson index are in the range from 0 (low variability) to 1–1/S, and S is the number of species in the community. The higher the diversity, the higher the value of the Simpson index.

## 5. Conclusions

The meadow cultivation of clovers and grasses in post-bog habitats is characterized by a high frequency and diversity of *Fusarium* communities colonizing the root zone of these plants. The semi-natural nature of the studied meadow plant community contributes to the selection of saprotrophic and endophytic strains in this group of fungi and the maintenance of the good health status of the plants.

## Figures and Tables

**Table 1:** Number of records (frequency) of fungi from the root zone of the analyzed plants isolated on the Nash and Snyder medium.

Complexes ^1^/Genera ^2^	Number of Records (Frequency)	% of Total Records
*Fusarium* ^1^	1447	82.9
*Cylindrocarpon* ^2^	6	0.3
*Ilyonectria* ^1^	27	1.6
*Fusicolla* ^2^	9	0.5
*Microdochium* ^2^	256	14.7
Total	1745	100.0

Explanations: ^1^: FSAMSC—*Fusarium sambucinum* species complex; FTSC—*Fusarium tricinctum* species complex; FIESC—*Fusarium incarnatum –equiseti* species complex; FOSC—*Fusarium oxysporum* species complex; FFSC—*Fusarium fujikuroi* species complex; FSSC—*Fusarium solani* species complex; FLSC—*Fusarium lateritium* species complex; FCAMSC—*Fusarium camptoceras* species complex; IRSC—*Ilyonectria radicicola* species complex.; ^2^: *Cylindrocarpon didymum; Cylindrocarpon magnusianum; Fusicolla aquaeductuum; Fusicolla merismoides; Microdochium nivale.*

**Table 2:** Total frequency of occurrence (in %) of the *Fusarium* and genera related to *Fusarium* in the clover and grass root zone.

Fungal Species ^1^/Complex ^2^	Non-Fertilized Clovers (nfC)		Fertilized Clovers (fC)		Non-Fertilized Grasses (nfC)		Fertilized Grasses (fG)		Total	
	Number of Isolates	%	Number of Isolates	%	Number of Isolates	%	Number of Isolates	%	Number of Isolates	%
*F. culmorum* (W.G. Smitch) Sacc. ^1^ FSAMSC ^2^	22	3.9	32	6.0	63	19.3	40	12.8	157	9.0
*F. graminearum* Schwabe FSAMSC	14	2.5	0	0.0	2	0.6	3	1.0	19	1.1
*F. poae* (Peck) Wollenw. FSAMSC	0	0.0	0	0.0	2	0.6	0	0.0	2	0.1
*F. sambucinum* Fuckel FSAMSC	33	5.8	22	4.1	8	2.4	6	1.9	69	3.9
*F. sporotrichioides* Sherb. FSAMSC	33	5.8	28	5.2	18	5.5	53	16.9	132	7.6
*F. avenaceum* (Fr.) Sacc. FTSC	98	17.2	39	7.3	23	7.0	40	12.8	200	11.4
*F. tricinctum* (Corda) Sacc. FTSC	1	0.2	0	0.0	0	0.0	0	0.0	1	0.1
*F. equiseti* (Corda) Sacc. FIESC	3	0.5	6	1.1	3	0.9	0	0.0	12	0.7
*F. incarnatum* (Desm.) Sacc. FIESC	0	0.0	0	0.0	1	0.3	4	1.3	5	0.3
*F. oxysporum* Schlecht. emand. Sny. and Hans. FOSC	241	42.3	302	56.2	128	39.1	100	31.9	771	44.1
*F. sacchari* (Butler) W. Gams FFSC	15	2.6	4	0.7	0	0.0	2	0.6	21	1.2
*F. solani* (Mart.) Sacc. FSSC	2	0.4	6	1.1	1	0.3	0	0.0	9	0.5
*F. lateritium* Nees ex Link FLSC	21	3.7	8	1.5	4	1.2	8	2.6	41	2.3
*F. camptoceras* Wollenw. & Reinking FCAMSC	1	0.2	4	0.7	2	0.6	1	0.3	8	0.5
*C. didymum* (Hartig) Wollenw.	0	0.0	0	0.0	1	0.3	3	1.0	4	0.2
*C. magnusianum* (Sacc.) Wollenw.	0	0.0	2	0.4	0	0.0	0	0.0	2	0.1
*Ilyonectria/Cylindrocarpon destructans* (Zinssm.) Rossman, L. Lombard and Crous IRSC	6	1.1	1	0.2	13	4.0	7	2.2	27	1.5
*Fusicolla aquaeductuum* (Radlk. and Rabenh.) Gräfenhan, Seifert and Schroers	0	0.0	2	0.4	1	0.3	5	1.6	8	0.5
*Fusicolla merismoides* (Corda) Gräfenhan, Seifert and Schroers	0	0.0	1	0.2	0	0.0	0	0.0	1	0.1
*Microdochium nivale* (Fr.) Samuels and I.C. Hallett	78	13.7	80	14.9	57	17.4	41	13.1	256	14.7
Total	570 a *	100	537 a	100	327 a	100	313 a	100	1747	100

Explanations: * homogenous groups: the same letters (a, b, c) indicate means that do not differ significantly from each other (at the significance level of α = 0.05); means of the compared pairs with different letters (e.g., a and b) differ significantly (at the same significance level of α = 0.05); FSAMSC—*Fusarium sambucinum* species complex; FTSC—*Fusarium tricinctum* species complex; FIESC—*Fusarium incarnatum–equiseti* species complex; FOSC—*Fusarium oxysporum* species complex; FFSC—*Fusarium fujikuroi* species complex; FSSC—*Fusarium solani* species complex; FLSC—*Fusarium lateritium* species complex; FCAMSC—*Fusarium camptoceras* species complex; IRSC—*Ilyonectria radicicola* species complex.

**Table 3:** Species dominance indices (D) of *Fusarium* and genera related to *Fusarium* in the rhizosphere and rhizoplane of non-fertilized and fertilized clovers and grasses—part 1 *.

C	Species ^1^/Complex ^2^	Ectorhizosphere (Ec)			Rhizoplane (Rp)			Endorhizosphere (Ed)			Total of Analyses		
		A_I_	A_II_	A_III_	A_I_	A_II_	A_III_	A_I_	A_II_	A_III_	E_c_	R_p_	E_d_
**nfC**	*F. sambucinum*^1^ FSAMSC ^2^	9.2	−	−	6.1	−	−	16.3	−	−	8.6	4.1	7.9
	*F. sporotrichioides* FSAMSC	9.2	−	−	13.6	−	−	11.6	2.9	−	8.6	9.2	6.7
	*Fusarium* *avenaceum* FTSC	29.2	−	−	12.1	3.8	−	13.9	2.9	−	27.1	9.2	7.9
	*F. oxysporum* FOSC	36.9	100.0	−	48.5	46.2	−	41.9	94.1	−	41.4	44.9	56.2
	*Microdochium nivale*	−	−	−	6.1	38.5	100.0	−	−	100.0	−	20.4	13.5
**nfG**	*F. culmorum* FSAMSC	33.3	33.3	50.0	11.1	16.7	−	−	−	20.0	38.5	11.6	4.3
	*F. poae* FSAMSC	6.7	−	12.5	−	−	−	−	−	−	7.7	−	−
	*F. sambucinum* FSAMSC	20.0	−	−	−	−	−	−	−	−	11.5	−	−
	*F. sporotrichioides* FSAMSC	−	−	−	11.1	−	−	25.0	−	−	−	7.0	10.0
	*F. avenaceum* FTSC	13.3	33.3	−	7.4	33.3	−	−	−	−	11.5	14.0	−
	*F. oxysporum* FOSC	20.0	−	−	44.4	16.7	−	53.6	100.0	6.7	11.5	32.6	61.4
	*F. camptoceras* FCAMSC	−	33.3	−	−	−	−	−	−	−	3.8	−	−
	*Ilyonectria/Cylindrocarpon destructans* IRSC	6.7	−	−	−	−	−	17.9	−	46.7	3.8	−	17.1
	*F. aquaeductuum*	−	−	12.5	−	−	−	−	−	−	3.8	−	−
	*Microdochium nivale*	−	−	25.0	14.8	33.3	100.0	−	−	26.7	7.7	27.9	5.7
**fC**	*F. sambucinum* FSAMSC	−	−	−	0.9	5.9	−	18.0	−	−	−	1.4	11.7
	*F. sporotrichioides* FSAMSC	23.3	2.9	18.2	−	11.8	−	−	−	−	13.3	1.4	−
	*F. avenaceum* FTSC	10.0	2.9	9.1	−	−	−	26.0	−	−	6.7	−	16.9
	*F. oxysporum* FOSC	53.3	82.4	63.6	85.2	58.8	42.9	46.0	96.3	−	68.0	79.9	63.6
	*F. aquaeductuum*	−	−	−	−	11.8	−	−	−	−	−	1.4	−
	*Microdochium nivale*	−	−	−	7.8	11.8	57.1	−	−	−	−	10.8	−
**fG**	*F. culmorum* FSAMSC	−	−	−	22.9	−	−	35.7	8.6	−	−	11.3	20.6
	*F. sambucinum* FSAMSC	−	−	−	5.7	−	−	3.6	−	−	−	2.8	1.6
	*F. sporotrichioides* FSAMSC	3.0	−	−	14.3	−	9.1	7.1	−	−	25.0	9.9	3.2
	*F. avenaceum* FTSC	24.0	36.4	−	2.9	14.3	13.6	32.1	−	−	27.8	8.5	14.3
	*F. oxysporum* FOSC	16.0	36.4	−	17.1	71.4	36.4	7.1	91.4	−	22.2	33.8	54.0
	*F. lateritium* FLSC	12.0	9.1	−	2.9	−	9.1	−	−	−	11.1	4.2	−
	*F. camptoceras* FCAMSC	4.0	−	−	−	−	−	−	−	−	2.8	−	−
	*Ilyonectria/Cylindrocarpon destructans* IRSC	−	−	−	−	−	−	14.3	−	−	−	−	6.3
	*Fusicolla aquaeductuum*	−	18.2	−	−	−	−	−	−	−	5.6	−	−
	*Microdochium nivale*	−	−	−	34.3	14.3	27.3	−	−	−	−	28.2	−

Explanation: nfC—non-fertilized clovers; fC—fertilized clovers; nfG—non-fertilized grasses; fG—fertilized grasses; A_I_, A_II_, A_III_—analyses I (19.05), II (22.07), III (30.09); C-combination; *—frequency classes: frequent, very frequent, numerous, very, “–”—not recorded; FSAMSC—*Fusarium sambucinum* species complex; FTSC—*Fusarium tricinctum* species complex; FIESC—*Fusarium incarnatum –equiseti* species complex; FOSC—*Fusarium oxysporum* species complex; FFSC—*Fusarium fujikuroi* species complex; FSSC—*Fusarium solani* species complex; FLSC—*Fusarium lateritium* species complex; FCAMSC—*Fusarium camptoceras* species complex; IRSC—*Ilyonectria radicicola* species complex.

**Table 4:** Species dominance indices (D) of *Fusarium* and genera related to *Fusarium* in the rhizosphere and rhizoplane of non-fertilized and fertilized clovers and grasses—part 2 *.

C	Species ^1^/Complex ^2^	Ectorhizosphere (Ec)			Rhizoplane (Rp)			Endorhizosphere (Ed)			Total of Analyses		
		A_I_	A_II_	A_III_	A_I_	A_II_	A_III_	A_I_	A_II_	A_III_	Ec	Rp	Ed
**nfC**	*F. culmorum*^1^ FSAMSC ^2^	4.6	−	−	6.1	−	−	4.7	−	−	4.3	4.1	2.2
	*F. graminearum* FSAMSC	1.5	−	−	1.5	7.7	−	2.3	−	−	1.4	3.1	1.1
	*F. equiseti* FIESC	−	−	−	3.0	−	−	−	−	−	−	2.0	−
	*F. lateritium* FLSC	−	−	−	3.0	3.8	−	7.0	−	−	−	3.1	3.4
	*F. camptoceras* FCAMSC	−	−	−	−	−	−	2.3	−	−	−	−	1.1
	*Ilyonectria/Cylindrocarpon destructans* IRSC	9.2	−	−	−	−	−	−	−	−	8.6	−	−
**nfG**	*F. graminearum* FSAMSC	−	−	−	−	−	−	3.6	−	−	−	−	1.4
	*F. equiseti* FIESC	−	−	−	7.4	−	−	−	−	−	−	4.7	−
	*F. incarnatum* FIESC	−	−	−	3.7	−	−	−	−	−	−	2.3	−
**fC**	*F. culmorum* FSAMSC	6.7	8.8	−	2.6	−	−	10.0	−	−	6.7	2.2	6.5
	*F. sacchari* FFSC	−	2.9	9.1	−	−	−	−	−	−	2.7	−	−
	*F. lateritium* FLSC	−	−	−	3.5	−	−	−	−	−	−	2.9	−
	*F. camptoceras* FCAMSC	6.7	−	−	−	−	−	−	−	−	2.7	−	−
	*Ilyonectria/Cylindrocarpon destructans* IRSC	−	−	−	−	−	−	−	3,7	−	−	−	1.3
**fG**	*F. incarnatum* FIESC	8.0	−	−	−	−	−	−	−	−	5.6	−	−
	*F. sacchari* FFSC	−	−	−	−	−	4.5	−	−	−	−	1.4	−

Explanations: *—frequency classes: sporadic, rare “–”—not recorded; nfC—non-fertilized clovers; fC—fertilized clovers; nfG—non-fertilized grasses; fG –fertilized grasses; A_I_, A_II_, A_III_—analyses I (19.05), II (22.07), III (30.09); C-combination; *—frequency classes: frequent, very frequent, numerous, very, “–”—not recorded; FSAMSC—*Fusarium sambucinum* species complex; FTSC—*Fusarium tricinctum* species complex; FIESC—*Fusarium incarnatum –equiseti* species complex; FOSC—*Fusarium oxysporum* species complex; FFSC—*Fusarium fujikuroi* species complex; FSSC—*Fusarium solani* species complex; FLSC—*Fusarium lateritium* species complex; FCAMSC—*Fusarium camptoceras* species complex; IRSC—*Ilyonectria radicicola* species complex.

**Table 5:** Values of species similarity indices (S expressed in %) of the *Fusarium* communities and genera related to *Fusarium* in the clover and grass root zone.

Combination	Compared Communities	Analyses (A)			Total of Analyses	Mean of Analyses
		I	II	III		
nfC	Ec—Rp	60.0	20.0	0.0	60.0	26.7
	Rp—Ed	63.6	33.3	100.0	72.7	65.7
	Ec—Ed	60.0	33.3	0.0	54.5	31.1
nfG	Ec—Rp	30.0	40.0	25.0	33.3	31.7
	Rp—Ed	22.2	25.0	25.0	44.4	24.1
	Ec—Ed	25.0	0.0	33.3	36.4	19.4
fC	Ec—Rp	25.0	25.0	20.0	30.0	23.3
	Rp—Ed	50.0	16.7	0.0	33.3	22.2
	Ec—Ed	50.0	16.7	0.0	37.5	22.2
fG	Ec—Rp	44.4	40.0	0.0	36.4	28.1
	Rp—Ed	62.5	25.0	0.0	55.6	29.2
	Ec—Ed	33.3	20.0	0.0	30.0	17.8

Explanations: nfC—non-fertilized clovers; fC—fertilized clovers; nfG—non-fertilized grasses; fG—fertilized grasses; Ec—ectorhizosphere, Rp—rhizoplane, Ed—endorhizosphere.

**Table 6:** Simpson species diversity indices (D) of fungi in the individual root zones of fertilized and non-fertilized clovers and grasses.

Plant Combination	Analysis (A)	Ectorhizosphere (Ec)	Rhizoplane (Rp)	Endorhizosphere (Ed)	Total of Analyses		
					Ec	Rp	Ed
nfC	I	0.750	0.719	0.766	0.731	0.734	0.650
	II	0.000	0.630	0.112			
	III	−	0.000	0.000			
nfG	I	0.782	0.743	0.617	0.796	0.776	0.578
	II	0.667	0.722	0.000			
	III	0.656	0.684	0.667			
fC	I	0.642	0.266	0.678	0.510	0.349	0.548
	II	0.311	0.609	0.071			
	III	0.545	0.490	−			
fG	I	0.765	0.776	0.737	0.792	0.774	0.640
	II	0.694	0.449	0.157			
	III	−	0.756	−			

Explanations: nfC—non-fertilized clovers; fC—fertilized clovers; nfG—non-fertilized grasses; fG—fertilized grasses; “–”—not recorded.

**Table 7:** Contingency table for the χ^2^ test and the dependence and strength of the relationship between the frequency of occurrence of *Fusarium* communities and genera related to *Fusarium* and the colonized biotope.

Fungal Species ^1^/Complex ^2^	Microenvironment						Total	
	Ectorhizosphere		Rhizoplane		Endorhizosphere			
	Number	%	Number	%	Number	%	Number	%
*F. culmorum*^1^ FSAMSC ^2^	4	0.5	0	0.0	1	0.1	5	0.6
*F. graminearum* FSAMSC	0	0.0	4	0.5	0	0.0	4	0.5
*F. poae* FSAMSC	2	0.2	0	0.0	0	0.0	2	0.2
*F. sambucinum* FSAMSC	9	1.1	8	0.9	17	2.0	34	4.0
*F. sporotrichioides* FSAMSC	25	2.9	21	2.5	15	1.8	61	7.1
*F. avenaceum* FTSC	3	0.4	2	0.2	2	0.2	7	0.7
*F. equiseti* FIESC	18	2.1	20	2.3	23	2.7	61	7.1
*F. incarnatum* FIESC	2	0.2	1	0.1	0	0.0	3	0.4
*F. oxysporum* FOSC	91	10.6	193	22.5	176	20.5	460	53.7
*F. sacchari* FFSC	2	0.2	1	0.1	0	0.0	3	0.4
*F. lateritium* FLSC	1	0.1	3	0.4	2	0.2	6	0.7
*F. camptoceras* FCAMSC	37	4.3	21	2.5	27	3.2	85	9.9
*Ilyonectria/Cylindrocarpon destructans* IRSC	2 *	0.2	67	7.8	16	1.9	85	9.9
*Fusicolla aquaeductuum*	7	0.8	0	0.0	17	2.0	24	2.8
*Microdochium nivale*	4	0.5	10	1.2	3	0.4	17	2.0
Total	207.0	24.2	351.0	41.0	299.0	34.9	857.0	100.0
Statistics			Value		Probability			
Pearson’s chi-square			329.55		<0.0001			
Likelihood-ratio chi-square			345.65		<0.0001			
Mantel–Haenszel chi-square			9.59		0.002			
Φ-Yule’s coefficient			0.43					
C-Pearson contingency coefficient			0.40					
Cramer’s V coefficient			0.22					

Explanations: *—number of isolations; FSAMSC—*Fusarium sambucinum* species complex; FTSC—*Fusarium tricinctum* species complex; FIESC—*Fusarium incarnatum–equiseti* species complex; FOSC—*Fusarium oxysporum* species complex; FFSC—*Fusarium fujikuroi* species complex; FSSC—*Fusarium solani* species complex; FLSC—*Fusarium lateritium* species complex; FCAMSC—*Fusarium camptoceras* species complex; IRSC—*Ilyonectria radicicola* species complex.

**Table 8:** Selected physical and chemical properties of peat-muck soil.

Combination	% Organic Matter	% N Total	mg CaO in 100 g of Soil	mg w 100 g of Soil Acc. to Egner		Milligram Equivalents Ca in 100 g of Soil	pH 9 (KCl)
				P_2_O_5_	K_2_O		
Non-fertilized soil	51.59	1.78	95.03	13.70	7.19	33.87	4.40
Fertilized soil	72.78	2.25	147.78	16.00	9.90	55.68	5.15

## Data Availability

All data is contained within the article and supplementary material.

## Supplementary Materials

The following are available online at https://www.mdpi.com/article/10.3390/pathogens11030341/s1, Table S1: Composition and abundance of *Fusarium* and *Cylindrocarpon*/*Ilyonectria* populations in the rhizosphere (ectorhizosphere and endorhizosphere) of clovers from the permanent meadow, Table S2: Composition and abundance of *Fusarium* sensu lato and *Cylindrocarpon*/*Ilyonectria* populations in the rhizoplane of clovers from the permanent meadow, Table S3: Composition and abundance of *Fusarium* sensu lato and *Cylindrocarpon*/*Ilyonectria* populations in the rhizosphere (ectorhizosphere and endorhizosphere) of grasses from the permanent meadow, Table S4: Composition and abundance of *Fusarium* sensu lato and *Cylindrocarpon*/*Ilyonectria* populations in the rhizoplane of grasses from the permanent meadow.
