# Supplementary material for: Occurrence and Distribution of Fusarium Communities in the Root Zone in a Post-Bog Permanent Meadow in Relation to Mineral Fertilization and Growing Seasons
Source: Pathogens. 2022 Mar 11;11(3):341. doi: 10.3390/pathogens11030341 (PMC8951497; doi:10.3390/pathogens11030341)
Supplement: Supplementary file 1 [file pathogens-11-00341-s001.zip › pathogens-1538662-supplementary.pdf]

Supplementary material to the full paper to Pathogens:

Occurrence and distribution of *Fusarium* communities in the root zone in a post-bog permanent meadow in relation to mineral fertilization and growing seasons

Teresa Korniłłowicz-Kowalska Teresa [1], Bernadeta Wojdyło-Kotwica [1,2], Justyna Bohacz [1]*, Michał Możejko[1]

[1]University of Life Sciences in Lublin, Faculty of Agrobioengineering, Department of Environmental Microbiology, Leszczyńskiego 7 Street, 20-069 Lublin, Poland

[2]Alab plus Research Laboratory, 11 Wyszyńskiego Street, 05-220 Zielonka, Poland

*Corresponding author:

Justyna Bohacz

Tel.: +48 815248105; fax: +48 815248106

e-mail address: justyna.bohacz@up.lublin.pl

ORCID Justyna Bohacz 0000-0002-6670-0793

Table S1. Composition and abundance of *Fusarium* and *Cylindrocarpon* /*Ilyonectria* populations in the rhizosphere (ectorhizosphere and endorhizosphere) of clovers from the permanent meadow.

**Fungal species [1] /complex [2]**	**Non-fertilized**						**Fertilized**						**Total**			
	AI		AII		AIII		AI		AII		AIII		Ec,nfC	Ed,nfC	Ec,fC	Ed,fC
	Ec,nfC	Ed,nfC	Ec,nfC	Ed,nfC	Ec,nfC	Ed,nfC	Ec,fC	Ed,fC	Ec,fC	Ed,fC	Ec,fC	Ed,fC				
F. culmorum [1] FSAMSC [2]	3	1	-	-	-	-	2	5	3	-	-	-	3	2	5	5
F. graminearum FSAMSC	1	`	-	-	-	-	-	-	-	-	-	-	1	1	-	-
F. sambucinum FSAMSC	6	7	-	-	-	-	-	-	-	-	-	-	6	7	-	9
F. sporotrichioides FSAMSC	6	5	-	1	-	-	7	-	1	-	2	-	6	6	10	-
F. avenaceum FTSC	19	6	-	1	-	-	3	13	1	-	1	-	19	7	5	13
F. oxysporum FOSC	24	18	5	32	-	-	16	23	28	26	7	-	29	50	51	49
F. sacchari FFSC	-	-	-	-	-	-	-	-	1	-	1	-	-	-	2	-
F. lateritium FLSC	-	3	-	-	-	-	-	-	-	-	-	-	-	3	-	-
F. camptoceras FCAMSC	-	`	-	-	-	-	2	-	-	-	-	-	-	1	2	-
Ilyonectria/Cylindrocarpon destructans IRSC	6*	-	-	-	-	-	-	-	-	1	-	-	6	-	-	1
Microdochium nivale	-	-	-	-	-	12	-	-	-	-	-	-	-	12	-	-
**Total**	**65**	**43**	**5**	**34**	**0**	**12**	**30**	**50**	**34**	**27**	**11**	**0**	**70**	**89**	**75**	**77**
	**311**												**311**			

Explanations: Ec,nfC – ectorhizosphere of non-fertilized clovers; Ec,fC – ectorhizosphere of fertilized clovers; Ed,nfC – endorhizosphere of non-fertilized clovers; Ed,fC – endorhizosphere of fertilized clovers; A_I, A_{II}, A_{III} – analyses I (19.05), II (22.07), III (30.09); * - number of strains; **"-"** – not detected; FSAMSC - *Fusarium sambucinum* species complex, FTSC - *Fusarium tricinctum* species complex, FOSC - *Fusarium oxysporum*, species complex FFSC - *Fusarium fujikuroi* species complex, FLSC - *Fusarium lateritium* species complex, FCAMSC - *Fusarium camptoceras* species complex, IRSC - *Ilyonectria radicicola* species complex

Table S2. Composition and abundance of *Fusarium* sensu lato and *Cylindrocarpon* /*Ilyonectria* populations in the rhizoplane of clovers from the permanent meadow.

**Fungal species [1] / complex [2]**	**Term of analyses**																			
	**I (19.05)**						**II (27.07)**						**III (30.09)**						**Total**	
	**1nfC**	**1fC**	**2nfC**	**2fC**	**3nfC**	**3fC**	**1nfC**	**1fC**	**2nfC**	**2fC**	**3nfC**	**3fC**	**1nfC**	**1fC**	**2nfC**	**2fC**	**3nfC**	**3fC**	**1-3nfC**	**1-3fC**
F. culmorum [1] FSAMSC [2]	2	8	6	8	4	3	1	-	2	-	-	-	1	-	1	3	-	-	17	22
F. graminearum FSAMSC	3	-	3	-	1	-	-	-	2	-	2	-	-	-	1	-	-	-	12	-
F. sambucinum FSAMSC	5	4	9	6	4	1	-	-	2	-	-	1	-	1	-	-	-	-	20	13
F. sporotrichioides FSAMSC	5	8	6	7	9	-	-	1	1	-	-	2	-	-	-	-	-	-	21	18
F. avenaceum FTSC	32	13	19	3	8	-	-	1	2	-	1	-	1	1	9	3	-	-	72	21
F. tricinctum FTSC	-	-	-	-	-	-	-	-	-	-	-	-	-	-	1	-	-	-	1	-
F. equiseti FIESC	1	-	-	2	2	-	-	-	-	-	-	-	-	-	-	4	-	-	3	6
F. oxysporum FOSC	40	36	34	28	32	98	10	12	21	11	12	10	3	3	10	1	-	3	162	202
F. sacchari FFSC	-	-	-	-	-	-	-	2	7	-	-	-	2	-	6	-	-	-	15	2
F. solani FSSC	2	1	-	5	-	-	-	-	-	-	-	-	-	-	-	-	-	-	2	6
F. lateritium FLSC	4	1	4	3	2	4	-	-	2	-	1	-	1	-	4	-	-	-	18	8
F. camptoceras FCAMSC	-	1	-	1	-	-	-	-	-	-	-	-	-	-	-	-	-	-	-	2
C. magnusianum	-	-	-	-	-	-	-	1*	-	-	-	-	-	1*	-	-	-	-	-	2
F. aquaeductuum	-	-	-	-	-	-	-	-	-	-	-	2	-	-	-	-	-	-	-	2
F. merismoides	-	-	-	-	-	-	-	1	-	-	-	-	-	-	-	-	-	-	-	1
Microdochium nivale	2	30	14	8	4	9	3	4	8	2	10	2	14	9	5	12	6	4	66	80
**Total**	**96**	**102**	**95**	**71**	**66**	**115**	**14**	**22**	**47**	**13**	**26**	**17**	**22**	**15**	**37**	**23**	**6**	**7**	**409**	**385**
	**794**																		**794**	

Explanations: 1nfC, 2nfC, 3nfC – rhizoplane of non-fertilized clovers, wash 1, 2, and 3, respectively; 1fC, 2fC, 3fC – rhizoplane of fertilized clovers, wash 1, 2, and 3, respectively; * - number of strains; "-" – not detected; FSAMSC - *Fusarium sambucinum* species complex, FTSC - *Fusarium tricinctum* species complex, FIESC - *Fusarium incarnatum –equiseti* species complex, FOSC - *Fusarium oxysporum* species complex, FFSC - *Fusarium fujikuroi* species complex, FSSC - *Fusarium solani* species complex, FLSC - *Fusarium lateritium* species complex, FCAMSC - *Fusarium camptoceras* species complex

Table S3. Composition and abundance of *Fusarium* sensu lato and *Cylindrocarpon* /*Ilyonectria* populations in the rhizosphere (ectorhizosphere and endorhizosphere) of grasses from the permanent meadow.

**Fungal species [1] / complex [2]**	**Non-fertilized**						**Fertilized**						**Total**			
	AI		AII		AIII		AI		AII		AIII					
	Ec,nfG	Ed,nfG	Ec,nfG	Ed,nfG	Ec,nfG	Ed,nfG	Ec,fG	Ed,fG	Ec,fG	Ed,fG	Ec,fG	Ed,fG	Ec,nfG	Ed,fG	Ec,fG	Ed,fG
F. culmorum [1] FSAMSC [2]	5	-	1		4	3	-	10	-	3	-	-	10	3	-	13
F. graminearum FSAMSC	-	1	-	-	-	-	-	-	-	-	-	-	-	1	-	-
F. poae FSAMSC	1	-	-	-	1	-	-	-	-	-	-	-	2	-	-	-
F. sambucinum FSAMSC	3	-	-	-	-	-	-	1	-	-	-	-	3	-	-	1
F. sporotrichioides FSAMSC	-	7	-	-	-	-	9	2	-	-	-	-	-	7	9	2
F. avenaceum FTSC	2	-	1	-	-	-	6	9	4	-	-	-	3	-	10	9
F. incarnatum FIESC	-	-	-	-	-	-	2	-	-	-	-	-	-	-	2	-
F. oxysporum FOSC	3	15	-	27	-	1	4	2	4	32	-	-	3	43	8	34
F. lateritium FLSC	-	-	-	-	-	-	3	-	1	-	-	-	-	-	4	-
F. camptoceras FCAMSC	-	-	1	-	-	-	1	-	-	-	-	-	1	-	1	-
Ilyonectria/Cylindrocarpon destructans IRSC	1*	5	-	-	-	7	-	4	-	-	-	-	1	12	-	4
Fusicolla aquaeductuum	-	-	-	-	1	-	-	-	2	-	-	-	1	-	2	-
Microdochium nivale	-	-	-	-	2	4	-	-	-	-	-	-	2	4	-	-
**Total**	**15**	**28**	**3**	**27**	**8**	**15**	**25**	**28**	**11**	**35**	**0**	**0**	**26**	**70**	**36**	**63**
	**195**												**195**			

Explanations: Ec,nfG– ectorhizosphere of non-fertilized grasses; Ec,fG– ectorhizosphere of fertilized grasses; Ed,nfG– endorhizosphere of non-fertilized grasses; Ed,fG – endorhizosphere of fertilized grasses; AI, AII, AIII – analyses I (19.05), II (22.07), III (30.09); * - number of strains; "-" – not detected; FSAMSC - *Fusarium sambucinum* species complex, FTSC - *Fusarium tricinctum* species complex, FIESC - *Fusarium incarnatum –equiseti* species complex, FOSC - *Fusarium oxysporum* species complex, FLSC - *Fusarium lateritium* species complex, FCAMSC - *Fusarium camptoceras* species complex, IRSC - *Ilyonectria radicicola* species complex

Table S4. Composition and abundance of *Fusarium* sensu lato and *Cylindrocarpon* /*Ilyonectria* populations in the rhizoplane of grasses from the permanent meadow.

**Fungal species [1]/ complex [2]**	**Term of analyses**																			
	**I (19.05)**						**II (27.07)**						**III (30.09)**						**Total**	
	**1nfG**	**1fG**	**2nfG**	**2fG**	**3nfG**	**3fG**	**1nfG**	**1fG**	**2nfG**	**2fG**	**3nfG**	**3fG**	**1nfG**	**1fG**	**2nfG**	**2fG**	**3nfG**	**3fG**	**1-3nfG**	**1-3fG**
F. culmorum [1] FSAMSC [2]	10	8	7	4	3	8	-	3	8	1	2	-	6	1	14	2	-	-	50	27
F. graminearum FSAMSC	-	2	-	-	-	-	-	-	1	-	-	-	-	-	-	1	-		1	3
F. sambucinum FSAMSC	4	2	-	1	-	2	1	-	-	-	-	-	-	-	-	-	-	-	5	5
F. sporotrichioides FSAMSC	3	12	4	13	3	5	-	1	1	1	-	-	-	7	-	1	-	2	11	42
F. avenaceum FTSC	5	6	-	2	2	1	2	1	2	1	4	2	4	1	1	4	-	3	20	21
F. equiseti FIESC	-	-	-	-	2	-	-	-	1	-	-	-	-	-	-	-	-	-	3	-
F. incarnatum FIESC	-	-	-	2	1	-	-	-	-	-	-	-	-	-	-	-	-	-	1	2
F. oxysporum FOSC	23	8	20	7	12	6	4	8	14	7	2	10	5	2	2	2	-	8	82	58
F. sacchari FFSC	-	-	-	-	-	-	-	-	-	1	-	-	-	-	-	-	-	1	-	2
F. solani FSSC	-	-	1	-	-	-	-	-	-	-	-	-	-	-	-	-	-	-	1	-
F. lateritium FLSC	2	-	2	-	-	1	-	-	-	-	-	-	-	-	-	1	-	2	4	4
F. camptoceras FCAMSC	-	-	1	-	-	-	-	-	-	-	-	-	-	-	-	-	-	-	1	-
C. didymum	-	3*	1	-	-	-	-	-	-	-	-	-	-	-	-	-	-	-	1	3
Ilyonectria/Cylindrocarpon destructans IRSC	-	-	-	-	-	-	-	1	-	2	-	-	-	-	-	-	-	-	-	3
Fusicolla aquaeductuum	-	-	-	-	-	-	-	3	-	-	-	-	-	-	-	-	-	-	-	3
Microdochium nivale	10	9	8	6	4	12	2	2	8	-	4	2	2	2	9	2	4	6	51	41
**Total**	**57**	**50**	**44**	**35**	**27**	**35**	**9**	**19**	**35**	**13**	**12**	**14**	**17**	**13**	**26**	**13**	**4**	**22**	**231**	**214**
	**445**																		**445**	

Explanations: 1nfG, 2nfG, 3nfG – rhizoplane of non-fertilized grasses, wash 1, 2, and 3, respectively; 1fG, 2fG, 3fG – rhizoplane of fertilized grasses, wash 1, 2, and 3, respectively; * - number of strains; **"-"** – not detected; FSAMSC - *Fusarium sambucinum* species complex, FTSC - *Fusarium tricinctum* species complex, FIESC - *Fusarium incarnatum –equiseti* species complex, FOSC - *Fusarium oxysporum* species complex, FFSC -

Fusarium fujikuroi species complex, FSSC - *Fusarium solani* species complex, FLSC - *Fusarium lateritium* species complex, FCAMSC - *Fusarium camptoceras* species complex, IRSC - *Ilyonectria radicicola* species complex
